# Extracellular Compounds from Pathogenic Bacterium *Pseudoalteromonas piscicida* X-8 Cause Bleaching Disease, Triggering Active Defense Responses in Commercially Farmed *Saccharina japonica*

**DOI:** 10.3390/biology12010047

**Published:** 2022-12-26

**Authors:** Yao Chen, Xiaoyang Zhang, Mingyu Ma, Yingrui Zhuang, Lirong Chang, Luyang Xiao, Gaoge Wang

**Affiliations:** 1 College of Marine Life Science, Ocean University of China, Qingdao 266003, China; 2 Institute of Evolution & Marine Biodiversity, Ocean University of China, Qingdao 266003, China; 3 Weihai Changqing Ocean Science & Technology Co., Ltd., Rongcheng 264316, China

**Keywords:** active defense responses, bleaching disease, *Saccharina japonica*, programmed cell death, *Pseudoalteromonas piscicida* X-8

## Abstract

**Simple Summary:**

Bleaching disease frequently occurs at the late nursery stage in the farmed brown alga *Saccharina japonica* with *Pseudoalteromonas piscicida* X-8 (PpX-8) having been identified as a pathogenic bacterium. However, whether PpX-8 can trigger active defense responses in *S. japonica* via programmed cell death still remains unknown. In this study, PpX-8 extracellular compounds were more pathogenic (*p* < 0.05) before being heated, indicating that the virulence factors of PpX-8 may exist in its extracellular compounds and that they are heat-sensitive. Based on the typical morphological changes and biochemical characteristics of 3′-OH ends resulting from DNA cleavage, as well as the enzymatic activity of caspase-3-like protein, PpX-8 extracellular compounds induced programmed cell death and thus triggered active defense responses in farmed *S. japonica*. The results of this work indicate that seaweeds and higher plants are conservative in their active defense responses when facing pathogenic bacterial infection, and lay the foundation for further investigation of the virulence mechanisms of PpX-8.

**Abstract:**

Pathogenic bacteria can trigger active defense responses in higher plants, leading to hypersensitive programmed cell death (PCD) to against those bacteria. However, related research on seaweeds is very limited. *Pseudoalteromonas piscicida* X-8 (PpX-8) has been identified as the pathogen that causes bleaching disease in commercially farmed *Saccharina japonica*. In this study, using an inoculation assay and microscopic observations, we found that the proportion of bleaching tissue pieces inoculated with PpX-8 extracellular compounds was significantly higher (*p* < 0.05) than that inoculated with heated extracellular compounds, indicating that the virulence factors of PpX-8 exist in extracellular compounds and they are heat-sensitive. Using TEM, we observed typical morphological characteristics of PCD after inoculation with extracellular compounds, including chloroplast shrinkage, cytoplasmic vacuolation, and intact mitochondrial structures. Moreover, we detected biochemical characteristics of PCD, such as 3′-OH ends resulting from DNA cleavage and caspase-3-like enzymatic activity, using a TUNEL assay and fluorescence staining. Therefore, PpX-8 extracellular compounds can induce PCD, thus triggering active defense responses in *S. japonica*. These results indicate that seaweeds and higher plants are conservative in their active defense responses against pathogenic bacteria. The results of this study lay the foundation for further investigation of the virulence mechanisms of PpX-8.

## 1. Introduction

When infected with pathogenic bacteria, higher plants can trigger active defense responses by inducing rapid cell death at the infection site to fight against the bacterial spread. This type of active defense response is referred to as a hypersensitive response (HR), and induced cell death during HRs is referred to as programmed cell death (PCD) [1,2,3,4,5,6,7,8]. Typical morphological characteristics of PCD mainly include chloroplast shrinkage, cytoplasmic vacuolation, and intact mitochondrial structures. Moreover, 3′-OH ends resulting from DNA cleavage and caspase-3-like enzymatic activity have also been detected in association with PCD [5,9,10,11,12,13,14,15,16].

Like higher plants, algae also display active defense responses, including PCD, after being infected with pathogenic viruses and bacteria. Caspase-like protein activity has been detected in the microalga *Emiliania huxleyi* after infection with the lytic virus (Ehv1), *Phaeobacter inhibens* DSM17395, or glycosphingolipids produced by the lytic virus (Ehv86), which indicate the occurrence of PCD [17,18,19]. In addition, PCD has also been documented in cultivated *Saccharina japonica* after infection with *Alteromonas macleodii*. Typical morphological characteristics of PCD, such as chloroplast shrinkage, cytoplasmic vacuolation, and intact mitochondrial structures, were observed in infected *S. japonica* [19]. Moreover, 3′-OH ends resulting from DNA cleavage and caspase-3-like enzymatic activity have been detected along with these morphological characteristics [20,21].

Worldwide, *S. japonica* is an important economic aquaculture seaweed [22,23,24,25,26,27]. Like higher crops, it suffers from various diseases both at the nursery stage and during field cultivation [28,29,30,31,32,33,34]. Bleaching disease, a bacterial disease, frequently occurs at the late nursery stage in farmed *S. japonica*, with disease symptoms including white and rotten sporeling tips [35]. If not well controlled, the bleached tips fall off the thalli, which seriously affects the supply of healthy sporelings. *Pseudoalteromonas piscicida* X-8 (PpX-8) was previously identified as a pathogenic bacterium for bleaching disease [35]. However, whether PpX-8 can trigger active defense responses in *S. japonica* via PCD remains unknown. Based on a *S. japonica*–PpX-8 experimental model, the aims of this study were (1) to investigate the pathogenicity of PpX-8 culture and various associated compounds, including intracellular compounds, extracellular compounds, heated extracellular compounds, and PpX-8 bacterial cells; (2) to observe microscopic and ultrastructural changes in *S. japonica* after inoculation with PpX-8 extracellular compounds; and (3) to detect 3′-OH ends resulting from DNA cleavage and caspase-3 enzymatic activity after inoculation with PpX-8 extracellular compounds. The results of this study can not only enrich the knowledge of algal pathology but can also provide a theoretical foundation for further work to elucidate the virulence mechanisms of PpX-8 and the defense response mechanisms of *S. japonica*.

## 2. Materials and Methods

### 2.1. Preparation of the Different Components of PpX-8

We prepared different components of PpX-8 by centrifuging PpX-8 culture, as described by Yu et al. [36] with some modifications. First, we thawed 1 mL of PpX-8 culture from storage at −80 °C and then added it to 100 mL of ZoBell 2216E liquid medium, followed by culturing at 25 °C and 120 rpm for 8 h. Then, we added 1 mL of PpX-8 culture to 100 mL of fresh ZoBell 2216E liquid medium and cultured it under the same conditions. We repeated this step three times. Then, we diluted 1 mL of the PpX-8 culture 10 times and inoculated it onto ZoBell 2216E marine agar plates using the streak plate method, and cultured plates at 25 °C until isolated colonies were formed. We inoculated single PpX-8 colony into 100 mL of ZoBell 2216E liquid medium and cultured them at 25 °C and 120 rpm for 8 h until the optical density (OD_600_) reached 0.5. Then, we centrifuged the PpX-8 culture at 8000× *g* for 30 min at 4 °C. We filtered the supernatant through a 0.22 μm Millipore^®^ cellulose membrane (Millipore Co., Ltd., Billerica, MA, USA) to prepare the extracellular compounds. We heated half of the extracellular compounds for 20 min at 90 °C and kept the other half at room temperature for further use. We prepared the PpX-8 bacterial cells by washing the PpX-8 cell precipitation three times with sterilized seawater. Finally, we broke the PpX-8 cells using the ultrasonic cell-break method at 300 W for 1 min 30 s and resuspended them in sterilized seawater to prepare the PpX-8 intracellular compounds.

### 2.2. Sample Collection

Healthy *S. japonica* juvenile sporophytes (30–40 cm in length) were freshly collected from Weihai Changqing Ocean Science & Technology Co., Ltd. (Rongcheng, Shandong province, China, 37°19′41″ N, 122°17′21″ E), in December 2020. Samples were placed into sterilized sealed bags and shipped to our laboratory with ice packs within 4 h. After their arrival at the laboratory, we immediately washed the juveniles with sterilized seawater and then cultured them in sterilized seawater in an incubator (10 ± 1 °C, 80 μEm^−2^ s^−1^, light:dark = 12 h:12 h) overnight. 

### 2.3. Inoculation Assay with Different Components of PpX-8

The inoculation assay included two control groups (ZoBell 2216E liquid medium and sterilized seawater) and five treatment groups, including PpX-8 culture, PpX-8 extracellular compounds, heated PpX-8 extracellular compounds, PpX-8 intracellular compounds, and PpX-8 bacterial cells. We washed the juvenile sporophytes of *S. japonica* with sterilized seawater at least three times. We cut the edges of healthy juvenile sporophytes into small tissue pieces (1.0 × 1.0 cm^2^) with a sterilized surgical blade. We randomly added each tissue piece to each well of a 24-well cell culture plate (Labselect, Beijing Labgic Technology Co., Ltd., Beijing, China). We added 1 mL of ZoBell 2216E liquid medium and 1 mL of sterilized seawater to the control group wells. We inoculated the treatment group wells with 1 mL of PpX-8 bacterial culture, PpX-8 extracellular compounds, heated PpX-8 extracellular compounds, PpX-8 intracellular compounds, and PpX-8 bacterial cells, respectively. The experiments included three time points: 4 h, 12 h, and 24 h. Each time point included four replicates, and we repeated the experiment three times. The infection conditions were as follows: 10 ± 1 °C, 80 μEm^−2^s^−1^, and light: dark = 12 h:12 h. We randomly collected the tissues at 4 h, 12 h, and 24 h and washed them with sterilized seawater three times. We observed morphological changes in the tissue pieces under a light microscope (Eclipse Ni, Nikon, Japan) and took photos using an imaging system (DS-Ri2, Nikon, Japan).

### 2.4. Statistical Proportions of Bleached Tissue Pieces Following Inoculation with Different Components of PpX-8 

The preparation of the different PpX-8 components, the inoculation assay with 30 biological replicates, and the culture conditions were the same as those described in Section 2.1 and Section 2.3. At 22 h after inoculation, we counted the number of tissue pieces with bleaching symptoms and calculated the proportion of bleached tissue pieces. We performed Shapiro–Wilk and Levene’s tests to test the normality of the data and the homogeneity of variances. Finally, we expressed the data as the means ± standard error of the mean (SEM) for our three independent experiments. After calculating the statistics of the overall difference using a one-way analysis of variance (ANOVA), we determined the statistics of differences between groups with Fisher’s least-significant difference (LSD) test in GraphPad Prism 8. *p* < 0.05 represented a significant difference, and *p* < 0.01 represented an extremely significant difference.

### 2.5. Morphological Observations of the S. japonica Tissue Pieces Inoculated with PpX-8 Extracellular Compounds 

We prepared the inoculation assay as described in Section 2.3 with some modifications. The inoculation assay included the control group inoculated with ZoBell 2216E liquid medium and the treatment groups inoculated with heated PpX-8 extracellular compounds and PpX-8 extracellular compounds. We randomly added tissue pieces (1.0 × 1.0 cm^2^) to each well of a 24-well cell culture plate (Labselect, Beijing Labgic Technology Co., Ltd., Beijing, China). We added 1 mL of ZoBell 2216E liquid medium to each of the control group wells. We added 1 mL of heated or unheated PpX-8 extracellular compounds to the treatment group wells, respectively. There were five time points: 6 h, 14 h, 22 h, 30 h, and 46 h. Each time point included four replicates, and we repeated the experiment three times. Tissues were randomly collected at 6 h, 14 h, 22 h, 30 h, and 46 h and were washed with sterilized seawater three times. After collection, we observed morphological changes in the tissues under a light microscope (Eclipse Ni, Nikon, Japan) and took photos using an imaging system (DS-Ri2, Nikon, Japan).

### 2.6. Ultrastructural Changes in the S. japonica Tissues Inoculated with PpX-8 Extracellular Compounds Observed under a Transmission Electron Microscope (TEM)

The inoculation assay was performed following the methods described in Section 2.5. We collected the tissue pieces at 6 h, 14 h, 22 h, 30 h, and 46 h and washed them with sterilized seawater three times before using them to determine any ultrastructural changes. Ultrathin sections were prepared as described by Zhang et al. [35]. First, the collected tissue pieces were treated with 1.5 mL of 2.5% glutaraldehyde and were stored at 4 °C for 4 h. After being rinsed with 0.1 M of phosphate-buffered saline (PBS, pH = 7.4) three times for 10 min each time, the samples were treated with 1% osmium tetroxide (OsO4) for 2 h at 40 °C. Then, samples were washed again with 0.1 M of PBS (pH = 7.4) three times for 10 min each time. The samples were immersed in a graded series of ethanol/water solutions (30%, 50%, 70%, 90%, and 100%) for 10 min each, and the 100% ethanol treatment was performed twice for 10 min each time. After that, the samples were treated with Spurr epoxy resin and polymerized at 37 °C, 45 °C and 65 °C, with 24 h at each temperature. After slicing samples into ultrathin sections, we immersed them in lead nitrate and uranyl acetate for 15 min each. Finally, we observed the ultrathin sections under the TEM (JEM-1200EX, JEOL, Japan). 

### 2.7. TdT-Mediated dUTP Nick-End Labeling (TUNEL) Assay

We used a TUNEL kit (G1501, Servicebio, Qingdao, China) to evaluate the 3′-OH ends resulting from DNA cleavage in *S. japonica* tissue pieces inoculated with PpX-8 extracellular compounds. The inoculation assay was conducted following the methods described in Section 2.5. We collected the tissue pieces at 6 h, 14 h, 22 h, 30 h, and 46 h and washed them with sterilized seawater three times. The paraffin sections were prepared by Servicebio (Qingdao, China). First, the collected tissue samples were fixed with Davidson’s AFA fixation solution for at least 24 h. Then, we dehydrated the tissues with a graded series of ethanol solutions and impregnated them with paraffin: 75% ethanol for 4 h; 85% ethanol for 2 h; 90% ethanol for 2 h; 95% ethanol for 1 h; anhydrous ethanol for 30 min, performed twice; alcohol benzene for 10 min; xylene for 10 min, performed twice; and 65 °C melted paraffin for 1 h, performed three times. After that, we transferred the tissues to an embedding box for embedding, after which we cut the tissues into 4 μm thick sections using a paraffin slicer. Then, we flattened the tissues with a spreading machine in a 40 °C water bath, loaded them onto glass slides, and dried them at 60 °C. 

TUNEL assay were carried out according to the manufacturer’s instructions with some modifications of TUNEL kit (G1501, Servicebio, Qingdao, China). First, the paraffin sections were dewaxed twice with xylene for 15 min each time, and twice with 95% ethanol for 10 min each time. Then, the slices were washed three times with distilled water for 5 min each time. Second, the slices were marked with an immunohistochemical pen and incubated them with Protease K (G1234, Servicebio, Qingdao, China; Protease K: PBS = 1:9) at 37 °C for 25 min. Then, the slices were washed with PBS (pH = 7.4) three times for 5 min each time. According to the number of slices and size of tissue pieces, we prepared the reaction mixture with terminal deoxynucleotidyl transferase (TDT) incubation buffer (recombinant TDT enzyme:FITC-12-dUTP labeling mixture:equilibrium buffer = 1:5:50), and we prepared the reaction mixture of the negative control with a ddH_2_O incubation buffer (ddH_2_O:FITC-12-dUTP labeled mixture:equilibrium buffer = 1:5:50). We treated the positive control with 100 μL of DNase I (4.6 U/ μL, Sigma-adlrich, Saint Louis, MO, USA). We added the reaction mixture to the slices and incubated them at 37 °C for 2 h. After that, the slices were washed three times with PBS (pH = 7.4) for 5 min each time and were sealed with an anti-fluorescence quenching agent. Finally, we examined the stained sections under fluorescence microscope (Eclipse50i, Nikon, Japan) and photographed them using an imaging system (NikonDS-U3, Nikon, Japan).

### 2.8. Detection of the Caspase-3-like Enzymatic Activity of S. japonica Tissues

We detected the caspase-3-like enzymatic activity of *S. japonica* tissues after inoculation with PpX-8 extracellular compounds using a CaspGLOWTM Fluorescein Active Caspase-3 Staining Kit (K183-25, BioVision, Milpitas, CA, USA). We performed the inoculation assay following the methods described in Section 2.3 with some modifications. The negative control was treated with the caspase-3-specific inhibitor benzyloxycarbonyl-valinyl-alaninyl-aspartyl fluoromethylketone (Z-VAD-FMK) after inoculation at 6 h and at 22 h. First, we collected the inoculated tissue pieces and washed them three times with sterilized seawater at 6 h, 14 h, 22 h, 30 h, and 46 h. Then, we placed the samples into a 1.5 mL Eppendorf centrifuge tube. After that, we added 1 mL of sterilized seawater and 1.2 μL of fluorescent tagged caspase-3 inhibitor (FITC-DEVD-FMK) to each centrifuge tube. The negative control was treated with caspase-3-specific inhibitor Z-VAD-FMK at 6 h and at 22 h. The tissue pieces were inoculated in an incubator containing 5% CO_2_ at 37 °C for 45–60 min. Then, the tissues were washed with sterilized seawater to remove excess FITC-DEVD-FMK. Finally, we observed caspase-3-like enzymatic activity under a fluorescence microscope (Eclipse 50 ineedle, Nikon, Japan) and photographed it using an imaging system (NikonDS-U3, Nikon, Japan). Green fluorescence signals represented caspase-3-like enzymatic activity, whereas no or weak green fluorescence signals in the cells represented no caspase-3-like enzymatic activity.

## 3. Results

### 3.1. Inoculation Assay of S. japonica Tissues with Different Compounds of PpX-8 

**Figure 1:**
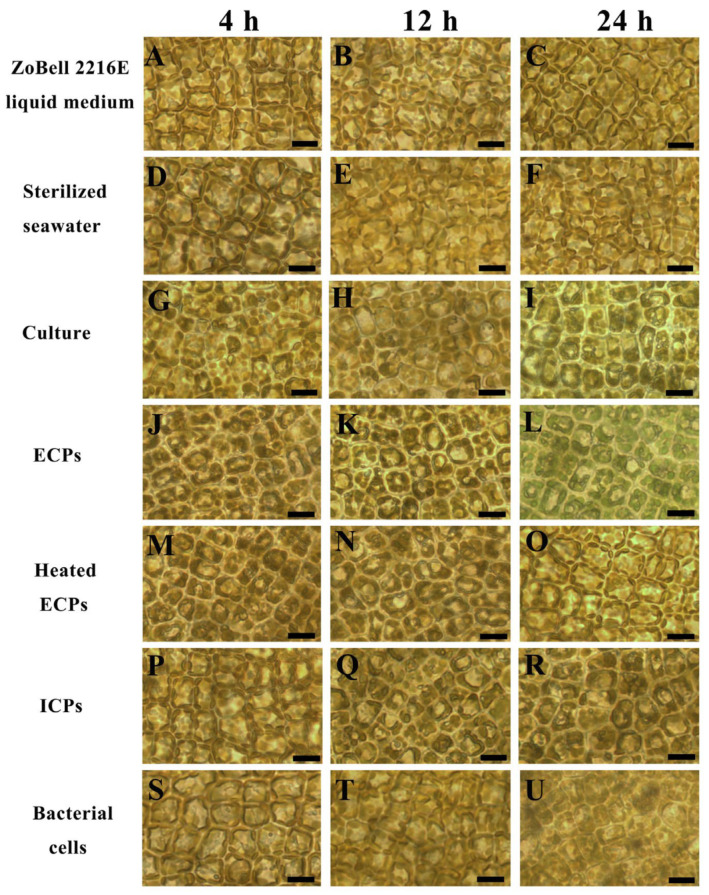
Inoculation assay of *S. japonica* tissue pieces with different PpX-8 components. (**A**–**C**): ZoBell 2216E liquid medium at 6 h, 12 h, and 24 h, respectively; (**D**–**F**): control groups inoculated with and sterilized seawater at 6 h, 12 h, and 24 h, respectively; (**G**–**I**): groups inoculated with PpX-8 culture at 6 h, 12 h, and 24 h, respectively; (**J**–**L**): groups inoculated with PpX-8 extracellular compounds at 6 h, 12 h, and 24 h, respectively; (**M**–**O**): groups inoculated with heated PpX-8 extracellular compounds at 6 h, 12 h, and 24 h, respectively; (**P**–**R**): groups inoculated with PpX-8 intracellular compounds at 6 h, 12 h, and 24 h, respectively; (**S**–**U**): groups inoculated with PpX-8 bacterial cells at 6 h, 12 h, and 24 h, respectively. Culture: PpX-8 culture; ECPs: PpX-8 extracellular compounds; Heated ECPs: heated PpX-8 extracellular compounds; ICPs: PpX-8 intracellular compounds. Bars: 50 μm.

In the inoculation assay, we inoculated tissue pieces from healthy juveniles with different compounds of PpX-8, including PpX-8 culture, heated PpX-8 extracellular compounds, PpX-8 extracellular compounds, PpX-8 intracellular compounds, and PpX-8 bacterial cells. Compared with the control group (Figure 1A,D) at 4 h, groups inoculated with PpX-8 culture (Figure 1G) and PpX-8 extracellular compounds (Figure 1J) showed mild bleaching symptoms, and no disease symptoms appeared in the groups inoculated with heated PpX-8 extracellular compounds (Figure 1M), PpX-8 intracellular compounds (Figure 1P), and PpX-8 bacterial cells (Figure 1S). Compared with the control group (Figure 1B,E), we observed significant bleaching symptoms at 12 h in groups inoculated with PpX-8 culture (Figure 1H) and extracellular compounds (Figure 1K). We observed mild bleaching symptoms in groups inoculated with heated PpX-8 extracellular compounds (Figure 1N), PpX-8 intracellular compounds (Figure 1Q), and PpX-8 bacterial cells (Figure 1T). Compared with the control group (Figure 1C,F), severe bleaching symptoms accompanied chloroplasts assembled around the cell wall at 24 h in the groups inoculated with PpX-8 culture (Figure 1I) and extracellular compounds (Figure 1L). We observed mild bleaching symptoms in the groups inoculated with heated PpX-8 extracellular compounds (Figure 1O), PpX-8 intracellular compounds (Figure 1R), and PpX-8 bacterial cells (Figure 1U).

### 3.2. Analysis of the Proportions of the Bleached Tissue Pieces 

**Figure 2:**
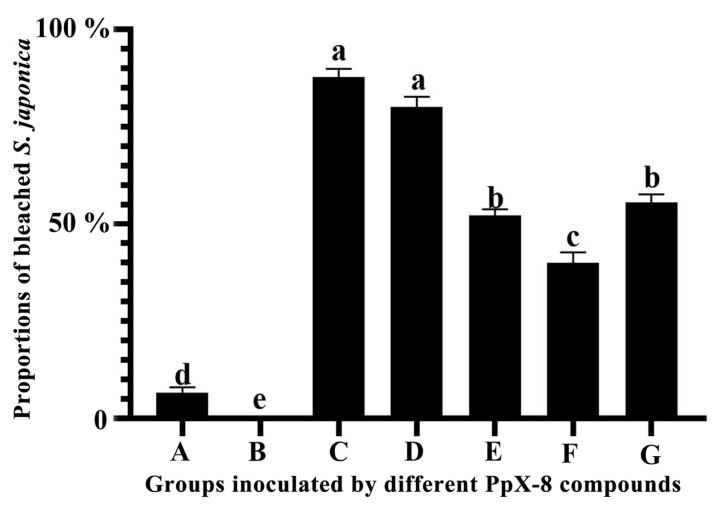
Proportion analysis of bleached pieces of *S. japonica* tissue after inoculation with different compounds of PpX-8. (**A**): control group inoculated with ZoBell 2216E liquid medium; (**B**): control group inoculated with sterilized seawater; (**C**): group inoculated with PpX-8 culture; (**D**): group inoculated with PpX-8 extracellular compounds; (**E**): group inoculated with PpX-8 heated extracellular compounds; (**F**): group inoculated with PpX-8 intracellular compounds; (**G**): group inoculated with PpX-8 bacterial cells. ANOVA (n = 30) showed a significant difference between groups inoculated with different components of PpX-8 (*p* < 0.05).

We calculated the proportions of bleached tissue pieces to determine the pathogenicity of the different PpX-8 compounds (Figure 2). Compared with the control groups inoculated with the 2216E liquid medium and sterilized seawater, the bleaching proportions at 22 h after inoculation was 87.8% and 80.0% for PpX-8 culture and PpX-8 extracellular compounds, respectively. The bleaching proportions were 40.0%, 52.2%, and 55.6% for the groups inoculated with PpX-8 intracellular compounds, heated PpX-8 extracellular compounds, and PpX-8 bacterial cells, respectively. We found no significant differences in the bleaching proportions (*p* > 0.05) between the groups inoculated with culture and with extracellular compounds. However, compared with other inoculated groups, the proportions were significantly different (*p* < 0.05).

### 3.3. Morphological Observations of S. japonica Tissue Pieces Inoculated with PpX-8 Extracellular Compounds

**Figure 3:**
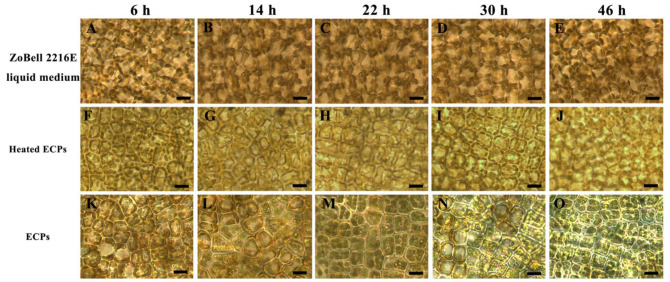
Microscopic changes in *S. japonica* tissue after inoculation with PpX-8 extracellular compounds. (**A**–**E**): control group inoculated with ZoBell 2216 liquid medium at 6 h, 14 h, 22 hand 46 h, respectively; (**F**–**J**): group inoculated with heated PpX-8 extracellular compounds at 6 h, 14 h, 22 hand 46 h, respectively; (**K**–**O**): group inoculated with PpX-8 extracellular compounds at 6 h, 14 h, 22 hand 46 h, respectively. ECPs: PpX-8 extracellular compounds at 6 h, 14 h, 22 hand 46 h, respectively; Heated ECPs: heated PpX-8 extracellular compounds at 6 h, 14 h, 22 hand 46 h, respectively. Bars: 50 μm.

Morphological images of *S. japonica* tissue pieces inoculated with PpX-8 extracellular compounds and heated PpX-8 extracellular compounds are shown in Figure 3. As shown in Figure 3, no bleaching symptoms appeared in the control group inoculated with ZoBell 2216E liquid medium at 6 h (Figure 3A), 14 h (Figure 3B), 22 h (Figure 3C), 30 h (Figure 3D), or 46 h (Figure 3E). In the group inoculated with PpX-8 extracellular compounds, bleaching symptoms gradually worsened with increasing inoculation time. Compared with the control group inoculated with ZoBell 2216E liquid medium at 6 h (Figure 3A) and 14 h (Figure 3B), mild bleaching symptoms appeared in the groups inoculated with heated PpX-8 extracellular compounds (Figure 3F,G) and PpX-8 extracellular compounds (Figure 3K,L). Compared with the control group (Figure 3C), we observed mild bleaching symptoms at 22 h in the group inoculated with heated PpX-8 extracellular compounds (Figure 3H), and obvious bleaching symptoms in the group inoculated with PpX-8 extracellular compounds (Figure 3M). Compared with the control group at 30 h (Figure 3D) and 46 h (Figure 3E), we observed obvious bleaching symptoms in the group inoculated with heated PpX-8 extracellular compounds (Figure 3I,J), and severe bleaching symptoms accompanied by the assembly of chloroplasts in the group inoculated with PpX-8 extracellular compounds (Figure 3N,O).

### 3.4. Ultrastructural Changes Observed under a Transmission Electron Microscope (TEM) in S. japonica Tissue Pieces Inoculated with Extracellular Compounds 

**Figure 4:**
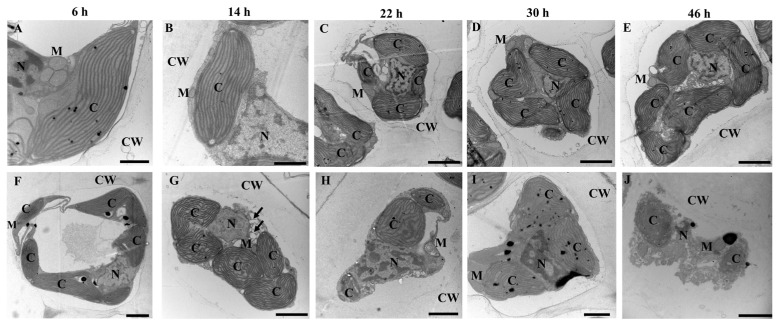
Ultrastructural changes in *S. japonica* inoculated with PpX-8 extracellular compounds. (**A**–**E**): control group inoculated with ZoBell 2216E liquid medium at 6 h, 14 h, 22 h, 30 h and 46 h, respectively; (**F**–**J**): group inoculated with PpX-8 extracellular compounds at 6 h, 14 h, 22 h, 30 h and 46 h, respectively. CW: cell wall; C: chloroplasts; M: mitochondria; N: nuclei. Black arrows indicate vacuolation. Bars in A and B: 1 μm; Bars in C, D, E, F, G, H, I, and J: 2 μm.

The ultrastructural changes in the *S. japonica* tissue pieces inoculated with PpX-8 extracellular compounds are shown in Figure 4. In the control group inoculated with ZoBell 2216E liquid medium (Figure 4A–E), the structures of chloroplasts, nuclei, mitochondria, and cell walls remained intact after inoculation. Compared with the control group at 6 h (Figure 4A), 14 h (Figure 4B), 22 h (Figure 4C), 30 h (Figure 4D), and 46 h (Figure 4E), no ultrastructural changes were observed in chloroplasts, nuclei mitochondria, and cell walls. In the group inoculated with PpX-8 extracellular compounds at 6 h (Figure 4F), the chloroplasts shrank and the cytoplasm vacuolated at 14 h (Figure 4G, arrow pointing to vacuolization). The nuclei began to deform, and the nuclear membranes began to dissolve at 22 h after inoculation (Figure 4H). Furthermore, the chloroplasts and their outer membranes began to dissolve, and the nuclei became fragmented at 30 h after inoculation (Figure 4I). The chloroplasts and the nuclei had disintegrated, and the mitochondrial outer membrane dissolved at 46 h (Figure 4J). The cell walls remained intact during the period of inoculation with PpX-8 extracellular compounds.

### 3.5. TUNEL Assay of S. japonica Tissue Pieces Inoculated with PpX-8 Extracellular Compounds 

**Figure 5:**
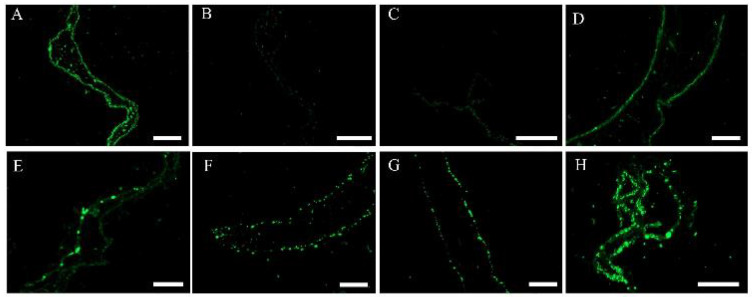
TUNEL detection results of *S. japonica* tissue pieces inoculated with PpX-8 extracellular compounds. (**A**): positive control; (**B**): negative control; (**C**): fluorescence signals in the control group inoculated with ZoBell 2216E liquid medium; (**D**–**H**): fluorescence signals exhibited in *S. japonica* tissue pieces inoculated with PpX-8 extracellular compounds at 6 h, 14 h, 22 h 30 h, and 46 h, respectively. Bars: 100 μm.

We used the TUNEL detection technique to detect 3′-OH ends resulting from DNA cleavage in *S. japonica* tissue pieces inoculated with PpX-8 extracellular compounds. Abundant fluorescence signals appeared in the positive control (Figure 5A), and no fluorescence signals appeared in the negative control (Figure 5B). Compared with the control inoculated with ZoBell 2216E liquid medium, the quantity of green fluorescence signals increased as the inoculation time increased (Figure 5D–H). In addition, we observed the most abundant green fluorescence signals at 46 h (Figure 5H).

### 3.6. Caspase-3-like Enzymatic Activity in S. japonica Tissue Pieces Inoculated with PpX-8 Extracellular Compounds

**Figure 6:**
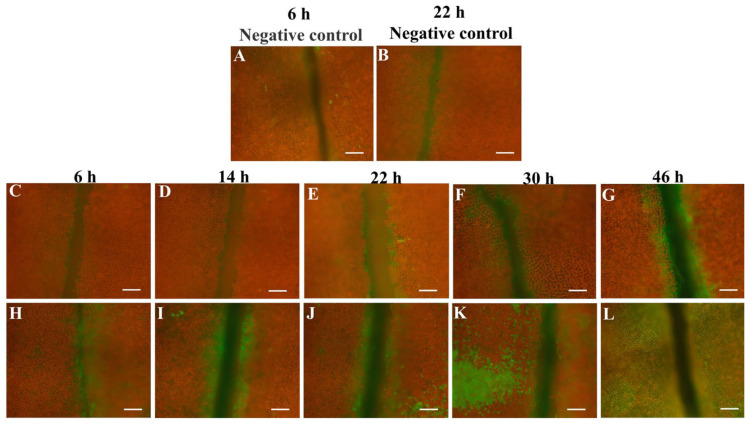
Results of caspase-3-like enzymatic activity detection of *S. japonica* tissue pieces inoculated with PpX-8 extracellular compounds. (**A**,**B**): negative control of *S. japonica* tissue pieces inoculated with Z-VAD-FMK treatment at 6 h and 22 h; (**C**–**G**): control inoculated with ZoBell 2216E liquid medium at 6 h, 14 h, 22 h, 30 h, and 46 h, respectively; (**H**–**L**): fluorescence signals exhibited in *S. japonica* tissue pieces inoculated with PpX-8 extracellular compounds at 6 h, 14 h, 22 h, 30 h, and 46 h, respectively. Bars: 100 μm.

Under treatment with the caspase-3-specific inhibitor Z-VAD-FMK, few green fluorescence signals appeared in the negative control at 6 h and 22 h (Figure 6A,B) after inoculation with PpX-8 extracellular compounds. Compared with the control group inoculated with ZoBell 2216E liquid medium at 6 h (Figure 6C), 14 h (Figure 6D), 22 h (Figure 6E), 30 h (Figure 6F), and 46 h (Figure 6G), green fluorescence signals appeared at 6 h after inoculation with PpX-8 extracellular compounds (Figure 6H). In addition, the intensity of green fluorescence signal increased as inoculation time increased (Figure 6H–L). At 30 h and 46 h, the green fluorescence signals disappeared at the inoculation sites but were transferred to the tissues, staying away from the inoculated sections and extending outward (Figure 6K,L). In addition, abundant green fluorescence signals appeared at 46 h (Figure 6L).

## 4. Discussion

In this study, we investigated the pathogenicity of different PpX-8 compounds based on a *S. japonica*–PpX-8 experimental model. The results showed that all the different PpX-8 compounds were pathogenic. However, the PpX-8 culture and extracellular compounds were the most pathogenic, with no significant difference found between them. Furthermore, the morphological and ultrastructural changes in *S. japonica* cells inoculated with PpX-8 extracellular compounds showed typical characteristics of PCD with 3′-OH ends resulting from DNA cleavage and caspase-3-like protein activity, indicating that PpX-8 extracellular compounds can induce PCD and trigger active defense responses in *S. japonica*.

The extracellular compounds of bacterial pathogens have been documented to be pathogenic in bacterial diseases. L-sucrase (EC2.4.1.10), secreted by *Pseudomonas syringae* pv. *phaseolicola*, pathogen of leaf spot disease of the yam bean *Pachyrhizus*, is an important virulence factor of extracellular enzymes, which can damage the recognition of the hosts at early stages of infection and can enhance pathogenicity [37]. *Pseudomonas syringae* pv. tomato DC 3000 (Psto) is the pathogen that causes tomato spot disease and can secrete the extracellular protein Cip1, which can inhibit the immune protein Pip1 in tomatoes and can promote the infection of Psto [38]. *Pseudomonas aeruginosa*, a pathogen that causes many human bacterial diseases, can produce a large number of extracellular compounds, including protease, alkaline protease, hemolysin, and exotoxin. These extracellular compounds are necessary for colonization and invasion [39,40,41,42,43]. In particular, *P. aeruginosa* can secrete two heat-sensitive phospholipases, including hemolytic phospholipase C (PLC-H) and nonhemolytic phospholipase C (PLC-N), which can promote *P. aeruginosa* infection [44]. In marine organisms, the extracellular compounds of various pathogenic bacteria have been identified. *Vibrio shiloi* is the pathogen for coral bleaching disease. PYPVYAPPPVVP, a polypeptide in the extracellular compounds of *V. shiloi*, can cause disintegration of the chloroplasts of *Zooxanthellae*, which is symbiotic with corals. Furthermore, the damaged chloroplasts affect the photosynthesis of *Zooxanthellae*, resulting in coral bleaching disease [45,46]. *Vibrio mediterranei* 117-T6 is the pathogen that causes yellow spot disease (YSD) in *Pyropia yezoensis*. The extracellular compounds of *V. mediterranei* 117-T6 can also lead to YSD, which shows the same symptoms as coral bleaching [47]. In this study, PpX-8 culture, PpX-8 extracellular compounds, heated PpX-8 extracellular compounds, PpX-8 intracellular compounds, and PpX-8 bacterial cells were found to be pathogenic. However, PpX-8 culture and PpX-8 extracellular compounds were the most pathogenic, and the pathogenicity was not significantly different between them, which indicates that the virulence factors of PpX-8 exist in its extracellular compounds. In addition, compared with the heated PpX-8 extracellular compounds, PpX-8 extracellular compounds were more pathogenic (*p* < 0.05), indicating that the virulence factors of PpX-8 are heat-sensitive. Therefore, we speculate that the virulence factors of PpX-8 exist in its extracellular compounds and are heat-sensitive. Further isolation and purification of PpX-8 extracellular compounds are needed in order to study their pathogenic mechanisms.

After being infected by pathogens, higher plants trigger their active defense responses through the HR, using PCD to prevent the further spread of pathogens. The typical morphological characteristics of PCD mainly include chloroplast shrinkage, cytoplasmic vacuolation, and intact mitochondrial structures [9,15,48,49,50]. One of the most important biochemical characteristics of PCD is the degradation of nuclear DNA, which can produce 3′-OH ends resulting from DNA cleavage [51,52,53,54,55,56,57,58,59]. Another biochemical characteristic of PCD is the activation of caspase-like protein activity [5,10,11,12,13,14,16,60,61]. Although no HR has been identified in seaweeds infected by pathogenic bacteria, PCD has been observed in seaweeds after such infection. Typical morphological characteristics of PCD were observed in commercially farmed *S. japonica* after infection with *A. macleodii* [20]. Moreover, 3′-OH ends resulting from DNA cleavage and caspase-3-like enzymatic activity have also been detected [20,21]. In this study, after inoculating tissue pieces from *S. japonica* with PpX-8 extracellular compounds, we observed typical morphological characteristics of PCD, such as chloroplast shrinkage, cytoplasmic vacuolation, and intact mitochondrial structures. We detected 3′-OH ends resulting from DNA cleavage and caspase-3-like enzymatic activity. This is similar to the reports of PCD during HRs in higher plants and in *S. japonica* infected with *A. macleodii*. PCD during HRs in higher plants involves the activation of specific endonucleases and the cascade action of caspase-like proteins [8,62,63,64,65], which are considered to be active defense responses against pathogens. The results of this work show that the extracellular compounds of PpX-8 can trigger active defense responses by inducing PCD in *S. japonica*. This indicates that seaweeds and higher plants are conservative in their active defense responses to fight against the pathogenic bacteria.

## 5. Conclusions

When infected with pathogenic bacteria, higher plants initiate active defense responses against bacterial infection through hypersensitive PCD. However, related research regarding commercially farmed seaweeds is still very limited. In this study, we found that the extracellular compounds of the pathogenic bacterium PpX-8 can trigger the active defense responses of farmed *S. japonica* through PCD. These results indicate that active defense responses in both seaweeds and higher plants are conservative when facing infection with pathogenic bacteria. Further studies are needed to isolate and purify the virulence factors in the extracellular compounds. The results of this study can not only help with understanding the active defense responses of *S. japonica* and with the facilitation of investigation into the virulence mechanisms of PpX-8, but also provide new insight into the pathology and active defense responses of seaweeds.

## Data Availability

The data presented in this study are available upon reasonable request from the corresponding authors. The data are not publicly available due to privacy.
